# The Prognostic Signature of Head and Neck Squamous Cell Carcinoma Constructed by Immune-Related RNA-Binding Proteins

**DOI:** 10.3389/fonc.2022.795781

**Published:** 2022-04-05

**Authors:** Ruijie Ming, Xiangrui Li, Enhao Wang, Jiahui Wei, Bo Liu, Peng Zhou, Wenting Yu, Shimin Zong, Hongjun Xiao

**Affiliations:** Department of Otorhinolaryngology, Union Hospital, Tongji Medical College, Huazhong University of Science and Technology, Wuhan, China

**Keywords:** head and neck squamous cell carcinoma, RNA binding protein, prognostic, immune microenvironment, tumor mutation burden, copy number variations, immunotherapy, chemotherapeutic

## Abstract

**Purpose:**

This study aimed to construct a prognostic signature consisting of immune-related RNA-binding proteins (RBPs) to predict the prognosis of patients with head and neck squamous cell carcinoma (HNSCC) effectively.

**Methods:**

The transcriptome and clinical data of HNSCC were downloaded from The Cancer Genome Atlas (TCGA) and Gene Expression Omnibus (GEO) databases. First, we ascertained the immunological differences in HNSCC, through single-sample gene set enrichment analysis, stromal and immune cells in malignant tumor tissues using expression data (ESTIMATE), and cell-type identification by estimating relative subsets of RNA transcripts (CIBERSORT) deconvolution algorithm. Then we used univariate proportional hazards (Cox) regression analysis and least absolute shrinkage and selection operator (LASSO) Cox regression analysis to screen immune-related RBPs and acquire the risk score of each sample. Subsequently, we further investigated the difference in prognosis, immune status, and tumor mutation burden in high- and low-risk groups. Finally, the efficacy of immunotherapy was measured by the tumor immune dysfunction and exclusion (TIDE) score.

**Results:**

We derived 15 immune-related RBPs, including FRMD4A, ASNS, RAB11FIP1, FAM120C, CFLAR, CTTN, PLEKHO1, SELENBP1, CHCHD2, NPM3, ATP2A3, CFDP1, IGF2BP2, NQO1, and DENND2D. There were significant differences in the prognoses of patients in the high- and low-risk groups in the training set (*p* < 0.001) and the validation set (*p* < 0.01). Furthermore, there were statistical differences between the high-risk group and low-risk group in immune cell infiltration and pathway and tumor mutation load (*p* < 0.001). In the end, we found that patients in the low-risk group were more sensitive to immunotherapy (*p* < 0.001), and then we screened 14 small-molecule chemotherapeutics with higher sensitivity to the high-risk group (*p* < 0.001).

**Conclusion:**

The study constructed a prognostic signature of HNSCC, which might guide clinical immunotherapy in the future.

## Introduction

Head and neck squamous carcinoma (HNSCC), which has a mortality rate of 50.5%, is one of the most common tumors, accounting for 3.6% of malignant tumors (1). HNSCC is a histologically and genetically heterogeneous disease that originates from a variety of anatomical parts, including the oral cavity, tongue, salivary glands, nasopharynx, and larynx (2). Smoking, drinking, and human papillomavirus infection are the main causes of HNSCC (3). Patients with HNSCC often experience cervical lymph node metastasis, local recurrence, and resistance to radiotherapy and chemotherapy (4).

At present, the treatment strategy for HNSCC patients is still based on tumor location and disease stage, not tumor biology. Many biomolecular markers, such as proteins, DNA, RNA, and microRNA, have been proposed to detect primary and secondary malignancies in the initial stages of the disease, but the above indicators are still very limited in terms of prognostic assessment and optimization of treatment options. In order to improve the treatment outcome of HNSCC, a clinically useful method is urgently needed to identify the risk of HNSCC and judge the effectiveness of adjuvant therapy.

The tumor microenvironment (TME) plays a vital role in the occurrence, progression, and treatment response of tumors. TME includes proliferating tumor cells, tumor stroma, blood vessels, cancer-related fibroblasts, infiltrating inflammatory cells, and various related signal molecules (5, 6). In the microenvironment of HNSCC, immune cells and mesenchymal cells, as the two main non-tumor components, have caused a large number of inflammatory reactions (7). Since HNSCC is an immunosuppressive disease, immune checkpoint inhibitors have emerged as a new treatment option (8). The basic principle of immunotherapy is to block the immunosuppressive effect of immune checkpoints while activating the endogenous immune system, thus increasing the number and cytotoxicity of T cells, which is beneficial to attack tumor cells (9). Consequently, it would be valuable to investigate the role of immune cells and their regulators in the TME of HNSCC.

From the nucleus to the peripheral cytoplasm, RNA-binding proteins (RBPs) play a vital role in the post-transcriptional regulation of genes (10). RBPs are able to affect pre-mRNA processing, transport and localization, mRNA stability/degradation, and translation (11). In a variety of tumors, some RBPs were found to be dysfunctional and aberrantly regulated (12, 13). Meanwhile, RBPs are important components of the immune system, which respond quickly to inflammatory mediators and in modulating inflammatory responses (14). Considering the important role of RBPs in immunity, it is necessary to explore the relationship between RBPs and HNSCC.

This study aimed to develop a prognostic prediction model for HNSCC based on immune-related RBPs. First, we classified HNSCC patients into two immune phenotypes based on the enrichment fraction of immune cells, then screened for differentially expressed RBPs in two immune phenotypes, and defined them as immune-related RBPs. Through univariate proportional hazards (Cox) regression analysis and least absolute shrinkage and selection operator (LASSO) Cox regression analysis, we identified immune-related RBPs related to prognosis and then constructed a risk model for patients with HNSCC. Based on the validation of the prognostic relevance and predictive capacity of the risk model, we further analyzed the infiltrating immune cells and immune-related pathways, somatic mutations, copy number variations (CNVs), the efficacy of immunotherapy, and sensitivity of chemotherapeutic agents in patients with HNSCC. The results showed that the risk model consisting of immune-related RBPs can effectively differentiate the clinical outcomes and show superiority in predicting the prognosis of patients with HNSCC.

## Methods

### Data Access

The transcriptome data in the fragment per kilobase million (FPKM) format and clinical data of 499 patients with HNSCC were downloaded from The Cancer Genome Atlas (TCGA) as the training set (https://portal.gdc.cancer.gov) (15) and downloaded the transcriptome data and clinical data of 97 HNSCC samples from the GSE41613 dataset of the Gene Expression Omnibus (GEO) database for validation (https://www.ncbi.nlm.nih.gov/geo/) (16). The data of somatic mutation and CNVs of patients with HNSCC were downloaded from UCSC (http://xena.ucsc.edu/) (17). The gene list of RBPs was collected from Gerstberger (10), SONAR (18), GO: RNA binding (19), poly(A) RBPs (20–24), CARIC (25), and XRNAX (26).

### Immunophenotyping Based on Single-Sample Gene Set Enrichment Analysis

Single-sample gene set enrichment analysis (ssGSEA) is an algorithm based on rank ordering, which can calculate the degree of enrichment of a single sample in a given gene set (27). On this basis, the enrichment scores of immune cells and some related immune processes were calculated through the GSEA program (28, 29) and then quantified through the default parameters of the “Gene Set Variation Analysis (GSVA)” R package (30). Subsequently, the “ConsensusClusterPlus” R package was used to co-cluster the infiltration levels of 23 types of immune cells in HNSCC samples from TCGA to identify and distinguish immune subtypes (31). In the cumulative distribution function (CDF), the K value with the largest area under the curve was selected as 2, and so the HNSCC samples were divided into two types (31). The Estimation of STromal and Immune cells in MAlignant Tumor tissues using Expression data (ESTIMATE) algorithm was utilized to calculate the immune score, stromal score, ESTIMATE score, and tumor purity (32). The immune cell infiltration calculated by the Cell-type Identification By Estimating Relative Subsets Of RNA Transcripts (CIBERSORT) deconvolution algorithm was used to verify the immune difference between the two types (33). Finally, the GSEA program was used to compare the differences in pathway enrichment between the above immunotypes from the Kyoto Encyclopedia of Genes and Genomes (KEGG) (34).

### Construction and Validation of Risk Model

The “limma” R package was used to distinguish RBPs with different expressions between immunotypes. With a 1.4-fold difference and corrected *p* less than 0.05 as the screening conditions, 238 immune-related RBPs were obtained. Subsequently, 47 immune-related RBPs associated with prognosis were obtained through univariate proportional hazards regression (*p* < 0.05). The “glmnet” package was then utilized to perform LASSO Cox regression analysis (35). After 1,000 times of cross-validation, 15 immune-related RBPs and the correlation coefficients of the corresponding risk genes were obtained to construct a risk model at the same time. $\text{Risk score}=\Sigma_{i}^{n}\;Expi\times Coefi$ , in which *Expi* is the expression of each risk gene and *Coefi* is its correlation coefficient. All patients were divided into a high-risk group and a low-risk group characterized by the median risk score of patients with HNSCC in the training set. The Kaplan–Meier curves were used to compare the overall survival (OS) difference of patients in the high- and low-risk groups. Receiver operating characteristic (ROC) curves were generated to evaluate the effectiveness and accuracy of the risk score in predicting the prognosis of patients with HNSCC. Next, the “ggExtra” R package was used to calculate the correlation between the risk score and the OS of patients with HNSCC. The independent correlation between the risk score and the prognosis of patients with HNSCC was then evaluated by univariate and multivariate proportional hazards regression analyses. Subsequently, a nomogram that could predict the prognosis of individual patients with HNSCC was constructed based on the stage, T stage, N stage, and risk group of patients with HNSCC through the “rms” R package (36). The C index was then used to assess the ability of the nomogram to distinguish prognosis, and a calibration chart was drawn to evaluate the accuracy of the nomogram. In addition, GSEA and gene set variation analysis (GSVA) were used to compare the differences in KEGG pathway enrichment between risk groups.

### Analysis of Somatic Mutation and Copy Number Variations

The tumor mutation burden (TMB) of HNSCC samples from TCGA was analyzed through the “maftools” R package (37). The differences in TMB between the high- and low-risk groups were compared and showed the top 20 genes with the highest mutation rate and their mutation types in the high- and low-risk groups. Then the impact of TMB on the OS of patients with HNSCC was evaluated through the Kaplan–Meier survival curves. After that, gistic 2.0 was used to detect significant copy number amplification or deletion (38). In the end, the CNVs of 22 pairs of autosomes between the high- and low-risk groups were compared and showed the top 20 genes with most CNVs and their variation types.

### Prediction of the Curative Effect of Immunotherapy and Chemotherapy

Tumor immune dysfunction and exclusion (TIDE) (http://tide.dfci.harvard.edu/) was used to calculate the TIDE score, which was reported to be able to predict the response of patients with a malignant tumor to immunotherapy (39). On the other hand, the “pRRophetic” R package was used to compare the half-maximal inhibitory concentration (IC50) differences of some common small-molecule chemotherapeutics between the high- and low-risk groups and screened out chemotherapeutics that may have better efficacy for patients in the high-risk group (40).

### Statistical Analysis

All statistical analyses were based on R 4.0.4 software (https://www.r-project.org/). Categorical variables were tested by the chi-square test or Fisher’s exact test. The t-test or Wilcoxon test was performed on continuous variables. *p* < 0.05 was deemed statistically significant.

## Results

### Development and Validation of the Prognostic Model Based on Immunophenotyping of Head and Neck Squamous Cell Carcinoma

**Figure 1:**
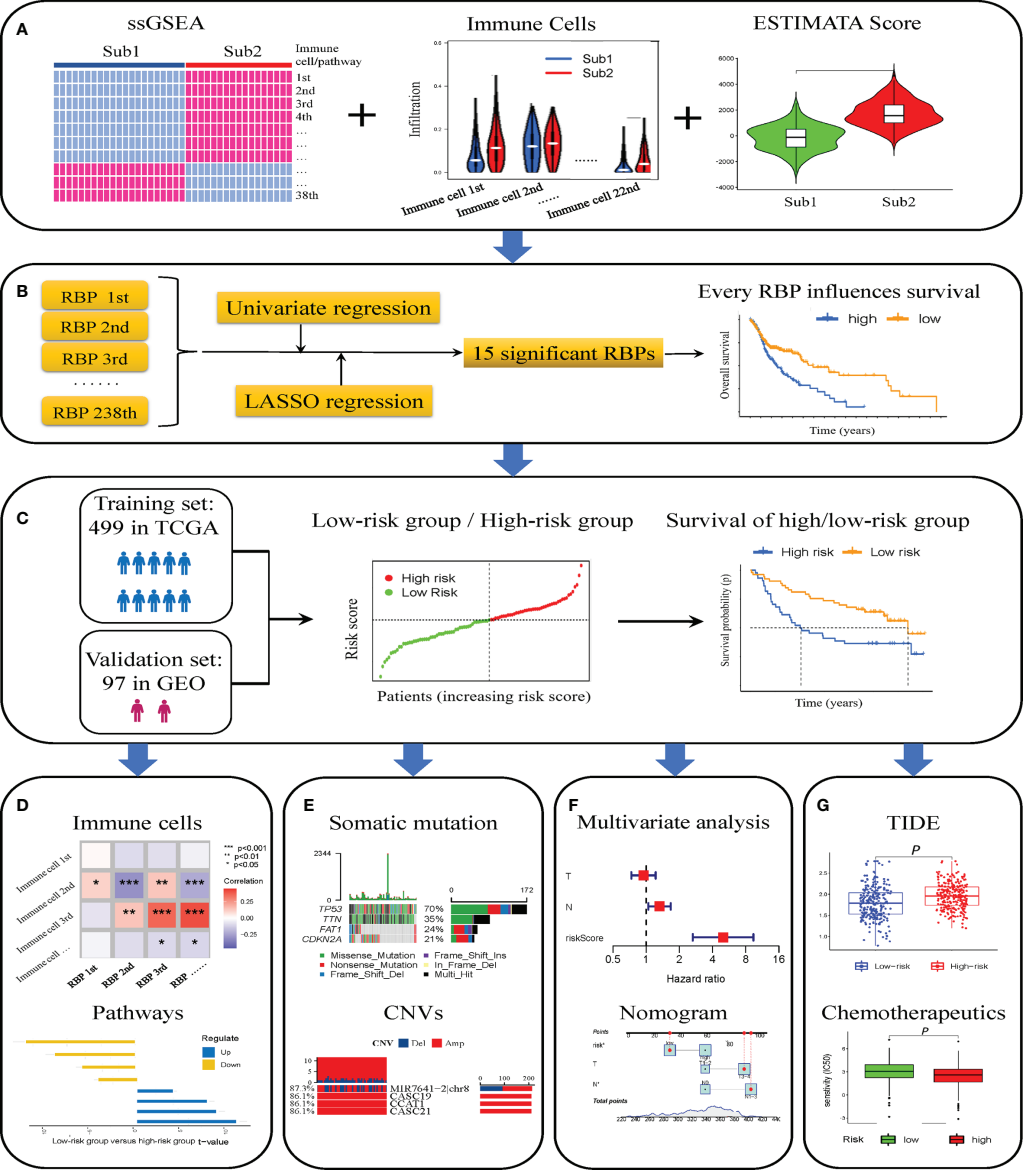
Flowchart of this study. Two immune subtypes identified by single-sample gene set enrichment analysis (ssGSEA) and co-clustering analysis, and difference of infiltrating immune cells assessed by CIBERSORT deconvolution algorithm and ESTIMATE algorithm **(A)**. Fifteen immune-related RNA-binding proteins (RBPs) screened out through “limma” package, univariate and least absolute shrinkage and selection operator (LASSO) Cox analysis, and the Kaplan–Meier curves for high- and low-expression immune-related RBP groups **(B)**. Validation of the risk model composed of immune-related RBPs for prognosis in The Cancer Genome Atlas (TCGA) and Gene Expression Omnibus (GEO) database **(C)**. Immune cell infiltration and pathways in high- and low-risk groups **(D)**. Somatic mutation and copy number variations (CNVs) in high- and low-risk groups **(E)**. Construction and calibration of prognosis nomogram **(F)**. The differences of tumor immune dysfunction and exclusion (TIDE) score and sensitivity to chemotherapeutics of patients with head and neck squamous cell carcinoma (HNSCC) in high- and low-risk groups **(G)**. *p < 0.05; **p < 0.01; ***p < 0.001.

The flowchart of this research is shown in **Figure 1**. First, we obtained patient data from TCGA database and divided the patients into two groups according to differences in immune cells. The CIBERSORT deconvolution and ESTIMATE algorithm confirmed the difference in the immune microenvironment between the Sub1 and Sub2 groups (**Figure 1A**). After differential expression analysis, 238 immune-related RBPs were identified. Through univariate and LASSO Cox regression analysis, 15 immune-related RBPs related to prognosis were selected, and then the Kaplan–Meier curves showed the difference between the high- and low-expression immune-related RBPs groups (**Figure 1B**). Subsequently, we found that the risk score was significantly related to the OS of patients with HNSCC in training and validation sets, respectively (**Figure 1C**). In addition, the differences in immune cells and pathways between the high- and low-risk groups are further elaborated (**Figure 1D**). In terms of genes, we showed the differences in somatic mutation and CNVs (**Figure 1E**). In addition, we have also produced a nomogram combining the stage, T stage, N stage, and risk group to predict the prognosis (**Figure 1F**). In the end, the efficacy of immunotherapy was analyzed through the TIDE score, and the sensitivity of different risk groups to small-molecule chemotherapeutics was also revealed (**Figure 1G**).

**Figure 2:**
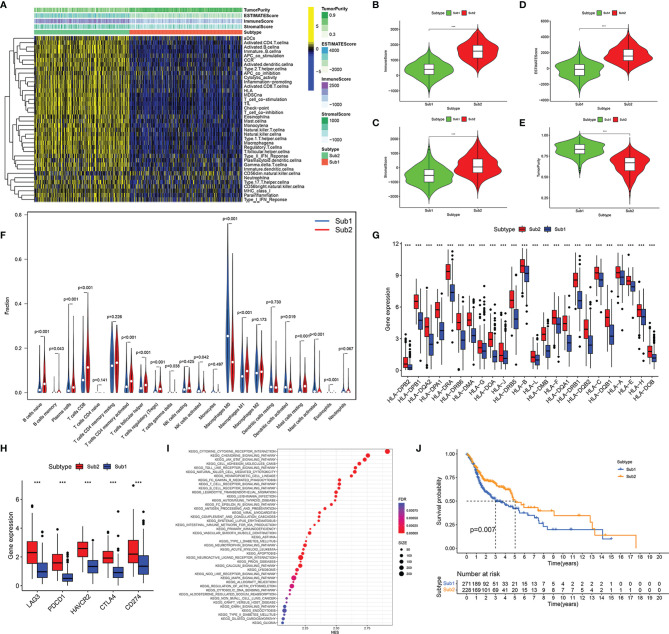
Immune subtypes of head and neck squamous cell carcinoma (HNSCC) were identified based on the tumor-infiltrating immune cells. Heatmap of single-sample gene set enrichment analysis (ssGSEA) scores for Sub1 group (n = 271) and Sub2 group (n = 228) **(A)**. Comparison of immune score **(B)**, stromal score **(C)**, ESTIMATE score **(D)**, and tumor purity **(E)** between Sub1 and Sub2 groups. Difference of immune cell infiltration between Sub1 and Sub2 groups **(F)**. The expressions of HLA family genes in Sub1 and Sub2 groups **(G)**. The discrepancy of immune checkpoint genes between Sub1 and Sub2 groups, including LAG3, PDCD1, HAVCR2, CTLA4, and CD274 **(H)**. The divergence of enrichment pathways between Sub1 and Sub2 groups **(I)**. Kaplan–Meier curves of Sub1 and Sub2 groups **(J)**. ***p < 0.001.

Based on the transcriptome data of TCGA HNSCC, we evaluated and quantify 23 kinds of immune cells and 15 kinds of immune processes by ssGSEA. After that, co-clustering analysis was used to distinguish the infiltration of 23 immune cells in the HNSCC samples in TCGA. When K = 2, the CDF curve had the largest area under the curve, so all samples were divided into two types (Sub1 and Sub2) (**Figure S1A**). Among them, there were 271 cases in the Sub1 group and 228 cases in the Sub2 group. It was worth mentioning that the immune cells and pathways were more enriched in the Sub2 group than the Sub1 group (**Figure 2A**). Compared with the Sub2 group, the Sub2 group had lower immune score (**Figure 2B**, *p* < 0.001), lower stromal score (**Figure 2C**, *p* < 0.001), lower ESTIMATE score (**Figure 2D**, *p* < 0.001), and higher tumor purity (**Figure 2E**, *p* < 0.001). For the purpose of authenticating the difference between the two types, we used the CIBERSORT deconvolution algorithm and the ESTIMATE algorithm to calculate the infiltration of immune cells. Among the Sub1 group, M0 macrophages, activated dendritic cells, and mast cells infiltrated more, while in the Sub2 group, primitive B cells, plasma cells, CD8 T cells, activated CD4 memory T cells, follicular helper T cells, Treg cells, M1 macrophages, resting mast cells, and eosinophils infiltrated more (**Figure 2F**, *p* < 0.05). As far as the human leukocyte antigen (HLA) family is concerned, the expression of the Sub1 group is lower (**Figure 2G**, *p* < 0.001). Considering the rise of immune checkpoint inhibitor therapy, we also analyzed the differences between immune checkpoints. The expressions of checkpoint LAG3, PDCD1, HAVCR2, CTLA4, and CD274 in the Sub2 group are extremely higher than that in the Sub1 group (**Figure 2H**, *p* < 0.001). In addition, as the result of pathway enrichment shows, there was more immune-related pathway enrichment in the Sub2 group, such as cytokine receptor interaction, chemokine signaling pathway, JAK-STAT signaling pathway, cell adhesion molecules cams, toll-like receptor signaling pathway, and natural killer cell-mediated cytotoxicity (**Figure 2I**, *p* < 0.001). It was worth noting that the Kaplan–Meier curves showed a better prognosis of the Sub2 group than that of the Sub1 group (**Figure 2J**, *p* = 0.007).

### Construction and Validation of Risk Model

**Figure 3:**
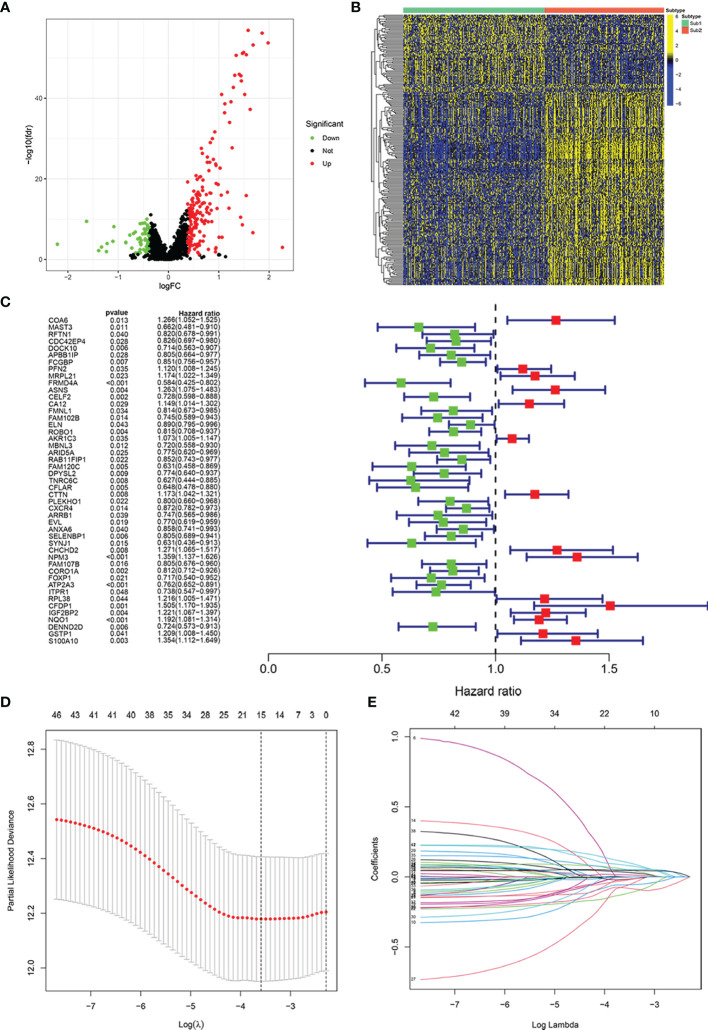
Construction of risk model for prognosis in patients with head and neck squamous cell carcinoma (HNSCC). Volcano plot exhibiting the differentially expressed immune-related RNA-binding proteins (RBPs) between Sub1 group (n = 271) and Sub2 group (n = 228) in HNSCC **(A)**. Heatmap of differentially expressed immune-related RBPs in Sub1 and Sub2 groups **(B)**. The result of univariate Cox analysis **(C)** and least absolute shrinkage and selection operator (LASSO) Cox analysis **(D, E)**.

We screened 238 immune-related RBPs through the “limma” R package (**Figure 3A**). Among these 238 immune-related RBPs, most of them were highly expressed in the Sub2 group, and the others were highly expressed in the Sub1 group (**Figure 3B**). Subsequently, 47 prognostic-related immune-related RBPs were obtained through univariate proportional hazards regression (**Figure 3C**, *p* < 0.05). In order to avoid overfitting, we then used LASSO Cox regression analysis and cross-validated 1,000 times to obtain 15 immune-related RBPs and the correlation coefficients of their corresponding risk genes (**Figures 3D, E**, **Table S1**). The risk model was thus constructed:


$$\begin{array}{l} \text{Risk Score}\;=Exp\text{FRMD}4\text{A}*(-0.0573)+\text{ExpASNS}*(0.1068)\\ +Exp\text{RAB}11\text{FIP}1*(0.1068)+Exp\text{FAM}120\text{C }*\;(-0.1220)\\ +\text{ ExpCFLAR }*\;(-0.0026)\;+\text{ExpCTTN }*\;(0.0341)\\ +\text{ ExpPLEKHO}1\;*\;(-0.0663)\;+\text{ ExpSELENBP}1\;*\text{ (0.0181)}\\ +\text{ ExpCHCHD}2\;*\;(0.0547)\;+\text{ ExpNPM}3\;*\;(0.0507)\\ +\text{ ExpATP}2\text{A}3\;*\;(0.0787)\;+\text{ ExpCFDP}1\;*\;(0.0787)\\ +\text{ ExpIGF}2\text{BP}2\;*\;(0.0149)+\text{ ExpNQO}1\;*\;(0.0459)\\ +\text{ ExpDENND}2\text{D }*\;(-0.0207). \end{array}$$

The negative correlation coefficient indicated that the expression of the gene was beneficial to the prognosis, and the positive value indicated no benefit or even hindrance.

**Figure 4:**
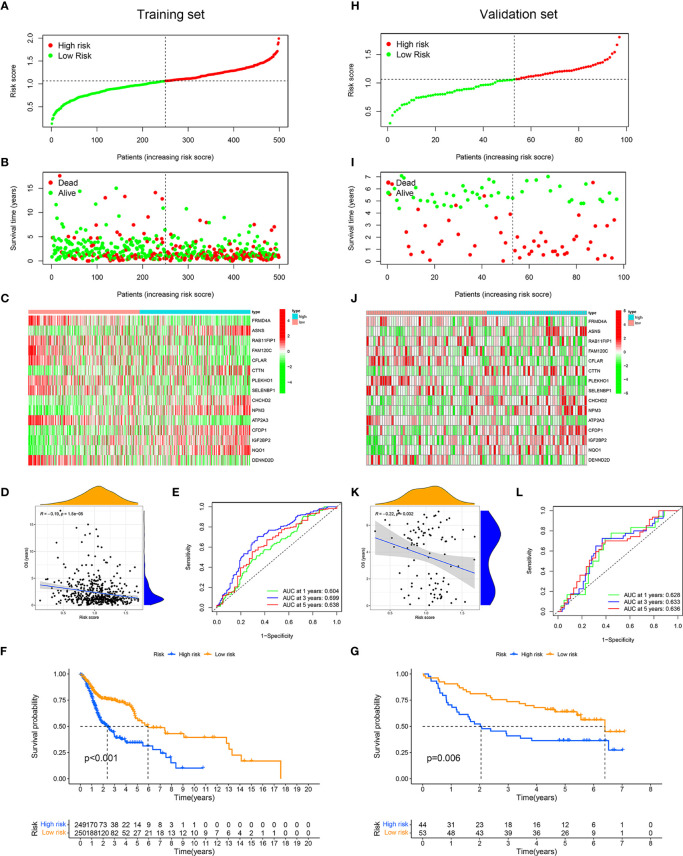
Application and validation of the risk model for prognosis. Samples in The Cancer Genome Atlas (TCGA) dataset were designated as training set, and samples in Gene Expression Omnibus (GEO) dataset were designated as validation set. On basis of the mean risk score of samples in training set, patients were divided into high-risk (red dot) and low-risk (green dot) groups. Distribution of the risk scores of the patients in training set **(A)**. Distribution of survival time of patients in training set **(B)**. The heatmap depicting the expression difference of 15 immune-related RNA-binding proteins (RBPs) between the high-risk group and the low-risk group in training set **(C)**. Correlation between overall survival and risk score in training set **(D)**. ROC curves of risk score for predicting 1, 3, and 5 years of overall survival in training set **(E)**. Kaplan–Meier curves of high- and low-risk groups in training set **(F)**. Distribution of the risk scores of the samples in validation set **(H)**. Distribution of survival time of samples in validation set **(I)**. The heatmap showing the expression patterns of 15 immune-related RBPs between the high- and low-risk groups in validation set **(J)**. Correlation between overall survival and risk score in validation set **(K)**. Receiver operating characteristic (ROC) curves of risk score for predicting 1, 3, and 5 years of overall survival in validation set **(L)**. Kaplan–Meier curves of high- and low-risk groups in validation set **(G)**.

We assigned TCGA data as the training set and GEO data as the validation set. According to the median risk score of patients with HNSCC in TCGA, all patients were divided into the high-risk group and low-risk group (**Figures 4A, H**). In the training and validation sets, the mortality of patients in the high-risk group was higher than that in the low-risk group (**Figures 4B, I**). In the high-risk group, ASNS, CTTN, CHCHD2, NPM3, CFDP1, IGF2BP2, and NQO1 were expressed higher, while in the low-risk group, there were higher expressions of FRMD4A, RAB11FIP1, FAM120C, CFLAR, PLEKHO1, SELENBP1, ATP2A3, and DENND2D (**Figures 4C, J**). The OS was negatively correlated with the risk score, which meant the OS of patients with HNSCC gradually decreases as the risk score increased (**Figures 4D, K**). The area under the ROC (AUC) of the risk score of the training set was 0.60 (1 year), 0.70 (3 years), and 0.64 (5 years) (**Figure 4E**). In contrast, the AUC of the validation set was 0.63 (1 year), 0.63 (3 years), and 0.64 (5 years) (**Figure 4L**). The Kaplan–Meier curves also indicated that the high-risk group had a poor prognosis (**Figures 4F, G**, *p* < 0.01).

**Figure 5:**
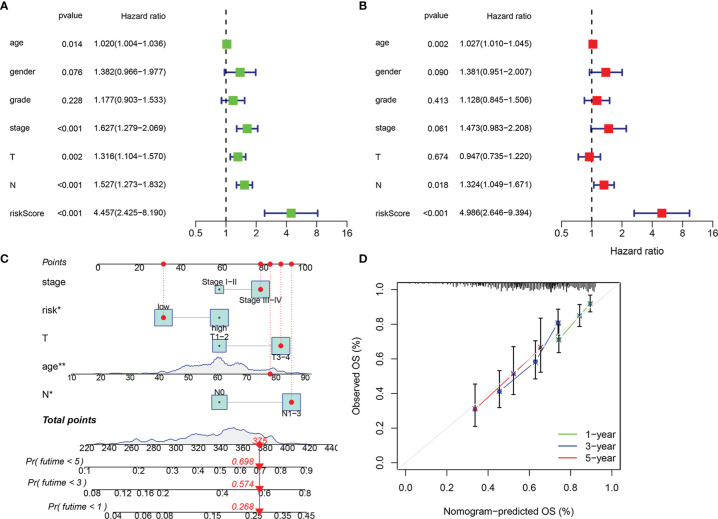
Independence of risk score and construction of nomogram consisting of risk score and clinicopathological characteristics. Univariate Cox regression analysis was used to validate whether age, gender, grade, stage, T, N, and risk score had an independent influence on prognosis **(A)**. Multivariate Cox regression analysis was used to validate whether age, gender, grade, stage, T, N, and risk score had independent influence on prognosis **(B)**. Construction of integrated nomogram to predict survival in head and neck squamous cell carcinoma (HNSCC) **(C)**. Calibration curve for predicting 1, 3, and 5 years of overall survival **(D)**. *p < 0.05, **p < 0.01.

In order to verify the validity and independence of the risk score, we combined the clinical characteristics and pathological staging data from TCGA database to perform univariate and multivariate Cox regression analyses. Univariate Cox analysis showed that age (*p* < 0.05), stage (*p* < 0.001), T (*p* < 0.01), N (*p* < 0.001), and risk score (*p* < 0.001) were significantly related to the prognosis (**Figure 5A**, **Table S2**). Multivariate analysis indicated that age (*p* < 0.01), N (*p* < 0.05), and risk score (*p* < 0.001) were significantly correlated with the prognosis (**Figure 5B**, **Table S3**). This implied that our risk model based on immune-related RBPs could be used as independent and effective indicators for the prognosis of patients with HNSCC.

In addition, we combined the stage (I–II and III–IV), T stage (T1–2 and T3–4), N stage (N0 and N1–3), and risk group (low and high) to construct 1-, 3-, and 5-year prognostic nomogram models (**Figure 5C**), which could guide clinical judgment more conveniently and effectively. For example, when an 80-year-old patient in a low-risk group is stage III–IV, T3–4, and N1–3, he would get a score of 375, which means that the probability of his survival time at less than 1 year, less than 3 years, and less than 5 years is 0.268, 0.574, and 0.698, respectively. The following calibration chart showed the difference between the OS predicted by this nomogram and the actual OS from TCGA database and suggested that the nomogram had certain accuracy (**Figure 5D**).

**Figure 6:**
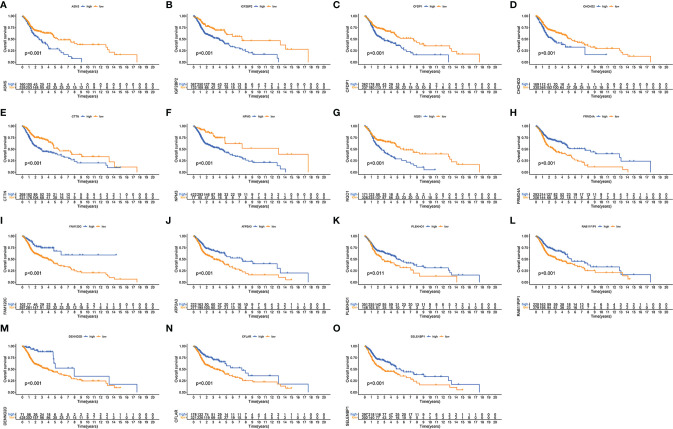
Validation of each immune-related RNA-binding protein (RBP) in the risk model. Kaplan–Meier curves showing the differences of overall survival in high- and low-expression immune-related RBPs ASNS **(A)**, IGF2BP2 **(B)**, CFDP1 **(C)**, CHCHD2 **(D)**, CTTN **(E)**, NPM3 **(F)**, NQO1 **(G)**, FRMD4A **(H)**, FAM120C **(I)**, ATP2A3 **(J)**, PLEKHO1 **(K)**, RAB11FIP1 **(L)**, DENND2D **(M)**, CFLAR **(N)**, and SELENBP1 **(O)** between high-expression (blue) group and low-expression (yellow) group.

Finally, we evaluated the relationship between each of the 15 immune-related RBP genes in the model and the OS of patients with HNSCC. Patients with high expressions of ASNS, IGF2BP2, CFDP1, CHCHD2, CTTN, NPM3, and NQO1 have poor OS, while patients with high expressions of FRMD4A, FAM120C, ATP2A3, PLEKHO1, RAB11FIP1, DENND2D, CFLAR, and SELENBP1 have a better OS (**Figures 6A–O**, *p* < 0.05).

### Exploration of the Immune Microenvironment

**Figure 7:**
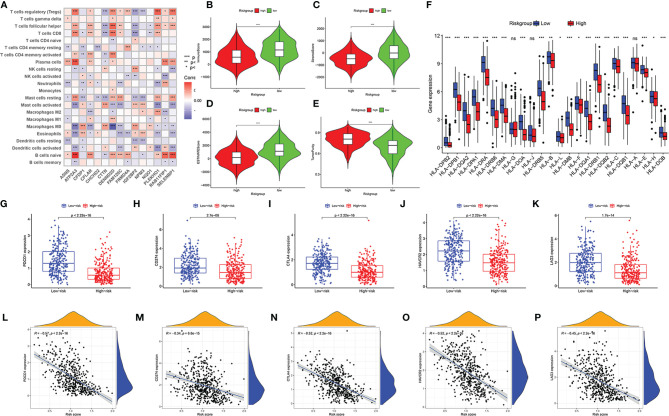
Immune landscape of patients with head and neck squamous cell carcinoma (HNSCC) in high- and low-risk groups. Correlation matrix of 15 immune-related RNA-binding proteins (RBPs) and infiltrating immune cells **(A)**. Comparison of immune score **(B)**, stromal score **(C)**, ESTIMATE score **(D)**, and tumor purity **(E)**. The differential expressions of HLA family genes in patients with HNSCC in high- and low-risk groups **(F)**. The expression level of immune checkpoint genes PDCD1 **(G)**, CD274 **(H)**, CTLA4 **(I)**, HAVCR2 **(J)**, and LAG3 **(K)** in low-risk group and high-risk group. The correlation between risk score and immune checkpoints PDCD1 **(L)**, CD274 **(M)**, CTLA4 **(N)**, HAVCR2 **(O)**, and LAG3 **(P)**. “ns” means p ≥ 0.05, *p < 0.05, **p < 0.01, ***p < 0.001.

The established risk model was based on immune-related RBPs, so it was necessary to confirm whether the model was related to the immune microenvironment of HNSCC. CIRBERSORT results showed that the 15 immune-related RBPs in the model all had associated immune cells (**Figure 7A**). Through the ESTIMATE algorithm, we found that the immune score, stromal score, and ESTIMATE score were lower and that the tumor purity was higher in the high-risk group (**Figures 7B–E**, *p* < 0.001). Then, we compared the expressions of the HLA family, and most of them were lower in the high-risk group (**Figure 7F**, *p* < 0.05). Subsequently, the checkpoint expressions of PDCD1, CD274, CTLA4, HAVCR2, and LAG3 in the low-risk group were relatively high (**Figures 7G–K**, *p* < 0.001). Every immune checkpoint is negatively correlated with the risk score (**Figures 7L–P**, *p* < 0.001).

### Analysis of Somatic Mutation and Copy Number Variations

**Figure 8:**
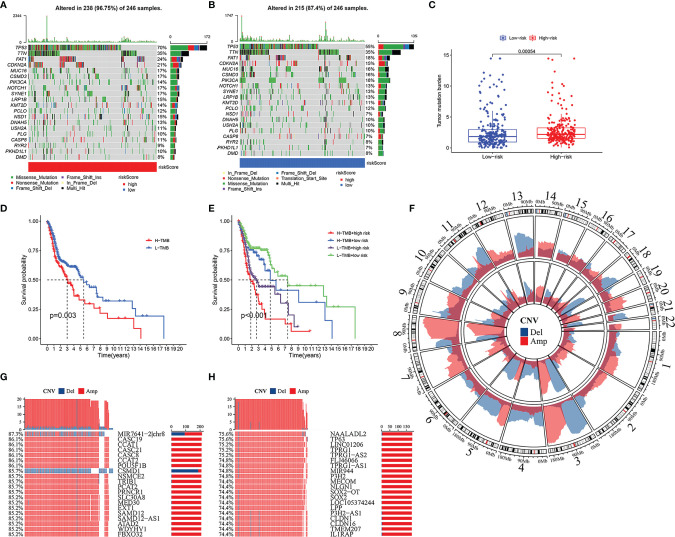
Somatic mutation and copy number variations (CNVs) in high- and low-risk groups. Heatmap of somatic mutations in high-risk group **(A)** and low-risk group **(B)**. The difference of tumor mutation burden between high- and low-risk groups **(C)**. Kaplan–Meier curves showing the differences in high- and low-tumor mutation burden (TMB) groups **(D)**. Kaplan–Meier curves revealing the differences in high-TMB and high-risk group, high-TMB and low-risk group, low-TMB and high-risk group, and low-TMB and low-risk group **(E)**. Amplification and deletion of copy number in the high-risk group (inner) and low-risk group (outer) **(F)**. The 20 genes with maximum CNVs in high-risk group, and the percentage meaning the proportion of patients with head and neck squamous cell carcinoma (HNSCC) who suffered gene deletion (blue) or amplification (red) in high-risk group **(G)**. Top 20 genes with maximum CNVs in low-risk group, and the percentage representing the ratio of patients with HNSCC who suffered gene deletion (blue) or amplification (red) in low-risk group **(H)**.

In the high-risk group and the low-risk group, the genes with the highest mutations are TP53, TTN, FAT1, and CDKN2A. Moreover, there are more mutations of TP53, FAT1, CDKN2A, NOTCH1, SYNE1, and NSD1 in the high-risk group, and the mutation rate of PIK3CA is higher in the low-risk group (**Figures 8A, B**). TMB is higher in the high-risk group (**Figure 8C**, *p* < 0.001). The prognosis of patients with high TMB was significantly worse than that of patients with low TMB (**Figure 8D**). Considering that the risk score was an independent prognostic factor, we evaluated the superimposed influence of TMB and risk score. The prognosis in descending order is the low-mutation and low-risk group, the high-mutation and low-risk group, the low-mutation and high-risk group, and the high-mutation and high-risk group (**Figure 8E**, *p* < 0.001).

Extensive copy number amplification was detected in 22 pairs of autosomes in all two groups. In the low-risk group, high-frequency deletion regions were found on chromosomes 3 and 13, and high-frequency amplification regions were found on chromosome 8 (**Figure 8F**). In the high-risk group, CNVs analysis indicated the following most relevant genes: MIR7641-2|chr8, CASC19, CCAT1, CASC21, CASC8, CCAT2, POU5FIB, and CSMD1 (**Figure 8G**). Among them, gene CSMD1 had a significant copy number deletion (**Figure 8G**). On the other hand, the five most correlative genes in the low-risk group included NAALADL2, TP63, LINC01206, TPRG1, and TPRG1-AS2 (**Figure 8H**).

**Figure 9:**
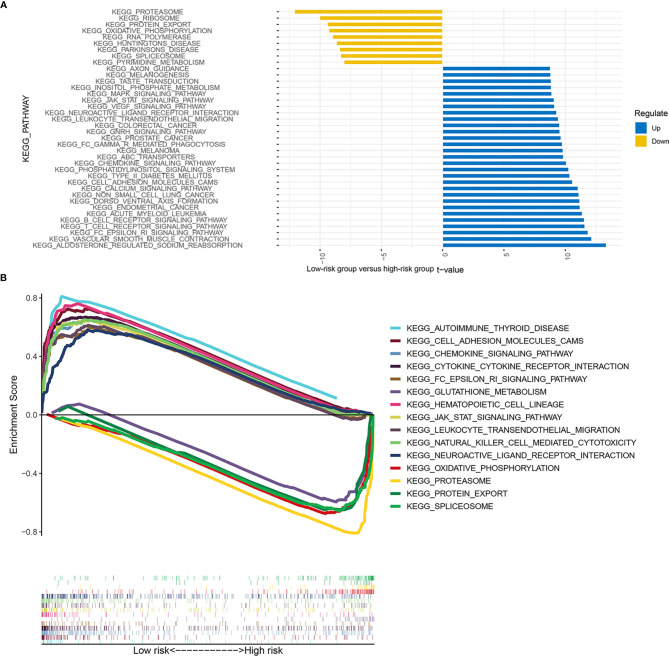
Enrichment signaling pathways of different risk groups. The pathway enrichment of gene set variation analysis (GSVA) between the low- and high-risk groups **(A)**. The pathway enrichment of gene set enrichment analysis (GSEA) between the low- and high-risk groups **(B)**.

GSEA (**Figure 9A**) and GSVA (**Figure 9B**) revealed the differences in pathway enrichment between the high- and low-risk groups. Most of the pathways enriched in the low-risk group were associated with immune responses, which may be involved in immune-related RBPs, including Fc gamma R-mediated phagocytosis, B-cell receptor signaling pathway, T-cell receptor signaling pathway, autoimmune thyroid disease, cell adhesion molecules cams, cytokine–cytokine receptor interaction, leukocyte transendothelial migration, and natural killer cell-mediated cytotoxicity.

### Prediction of the Efficacy of Immunotherapy and Chemotherapy

**Figure 10:**
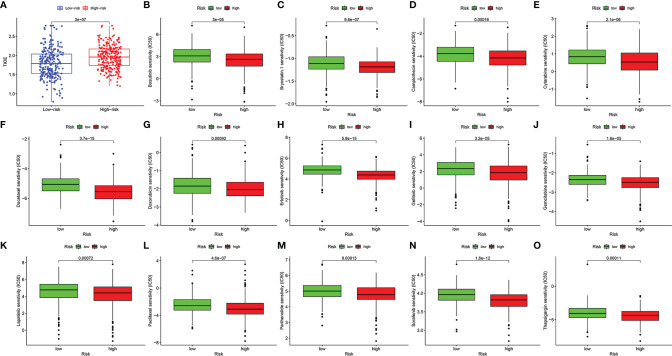
The value of the risk model in predicting the efficacy of immunotherapy and chemotherapy. The score of tumor immune dysfunction and exclusion of patients with head and neck squamous cell carcinoma (HNSCC) in high- and low-risk groups **(A)**. The box plots of the estimated IC50 for bosutinib **(B)**, bryostatin.1 **(C)**, camptothecin **(D)**, cytarabine **(E)**, docetaxel **(F)**, doxorubicin **(G)**, erlotinib **(H)**, gefitinib **(I)**, gemcitabine **(J)**, lapatinib **(K)**, paclitaxel **(L)**, parthenolide **(M)**, sorafenib **(N),** and thapsigargin **(O)**.

We used the TIDE score to predict the immunotherapy response of patients with HNSCC to immunotherapy. It could be briefly described that the higher the TIDE score, the higher the likelihood of immune dysfunction or evasion, and the less likely the patient will benefit from immune checkpoint inhibitors. As a result, the TIDE score of the high-risk group was significantly higher than that of the low-risk group, which means that immunotherapy is less effective in the high-risk group (**Figure 10A**, *p* < 0.001).

In addition, we screened out 14 small-molecule chemotherapeutics that may be more effective for patients with HNSCC in the high-risk group. The IC50 represents the concentration of an inhibitor that is required for 50% inhibition of carcinoma cells. A lower IC50 value means better drug sensitivity. Patients in the high-risk group were more sensitive to bosutinib, bryostatin.1, camptothecin, cytarabine, docetaxel, doxorubicin, erlotinib, gefitinib, gemcitabine, lapatinib, paclitaxel, parthenolide, sorafenib, and thapsigargin (**Figures 10B–O**, *p* < 0.001).

## Discussion

Immunotherapy has become an effective method for treating malignant tumors (41). Furthermore, immunosuppressant therapy has made important progress in the treatment of patients with HNSCC (42). Nevertheless, it cannot be ignored that only a limited one-third of patients respond to immunotherapy in most types of tumors (43). Further studies of immune-related RBPs in HNSCC may provide new ways to improve the clinical prognosis of patients. At present, there is an urgent need for an accurate and operational prognostic evaluation model for HNSCC in clinical practice. Based on TCGA and GEO databases and a variety of algorithms starting with ssGSEA, our study established a new model for predicting immune response, efficacy of conventional chemotherapy and immunotherapy, and individual outcome.

There are many kinds of myeloid immune cells in the HNSCC microenvironment that have a unique immune profile prior to treatment (44). In this study, we retrospectively analyzed the transcriptomic data of 499 HNSCC patients in TCGA database and further classified them into Sub1 and Sub2 on the basis of differences in immune cell infiltration. Regarding the infiltrating immune cells in the Sub1 group, M0 macrophages infiltrated more, while in the Sub2 group, there was more infiltration of naive B cells, plasma cells, T cells, and M1 macrophages. Compared with the Sub1 group, the Sub2 group had higher immune, stromal, and ESTIMATE scores but lower tumor purity, and its prognosis was significantly better than that of the Sub1 group. In addition, the expressions of the HLA family were significantly lower in the Sub1 group, which assisted tumor cells to escape the immune system (45). The immune-related pathways in the Sub2 group were more abundant.

Some RBPs are able to rapidly react to inflammatory mediators and regulate the reprogramming of immune cells to tumor-associated phenotypes (12). After recognizing the difference in RBP expressions between the Sub1 and Sub2 groups, we constructed a risk model containing 15 immune-related RBPs through univariate and LASSO Cox regression analysis. Then, according to the risk score calculated by the above model, patients with HNSCC were divided into low- and high-risk groups. Patients in the high-risk group had poorer clinical outcomes. The model even had good validity and stability in determining the prognosis at 1, 3, and 5 years, which was further confirmed in the GEO database. After confirming the risk score as an independent prognostic factor, we constructed a prognostic nomogram model according to the staging, T, N, and age of patients, which was also accurate in predicting OS at 1, 3, and 5 years.

The relationship between the prognostic characteristics of 15 immune-related RBPs and the immune microenvironment has also been investigated. In contrast with the high-risk group, the low-risk group had lower tumor purity and higher immune score, stromal score, and ESTIMATE score. In the high-risk group, M0 macrophages, activated dendritic cells, and mast cells infiltrated more, but the expression of the HLA family decreased. In the low-risk group, there were more M1 macrophages, naive B cells, CD4 memory and CD8 T cells, plasma cells, and eosinophils. This is similar to previous reports suggesting that exhausted immunity with lower survival is characterized by enrichment of stromal activation and anti-inflammatory M2 macrophage, whereas enhanced immunity associated with better prognosis is characterized by M1 macrophages providing stronger pro-inflammatory signaling, enhanced cytolytic activity, and massive lymphocyte infiltration (7). The activation of M1 macrophages is beneficial to patients because it can induce acute inflammation secreting tumor-killing molecules such as tumor necrosis factor α (TNFα) (46). On the other hand, if acute inflammation is not controlled, differentiation of M2 macrophages facilitates chronic inflammation, promoting tumor cell growth, angiogenesis, fibrosis, and immunosuppression (47), which is certainly harmful to patients. Both B cells and partial T cells also contribute to the prognosis of patients with HNSCC. As reported by Norouzian et al., the composition of B-cell subpopulations changes in TME of HNSCC, and the B cells with atypical memory and regulatory phenotype are significantly related to favorable prognostic (48). Notably, the high abundance of tumor-infiltrating lymphocyte B and high density of direct B-cell/CD8+ T-cell interactions predict a better outcome (49). Dense T-cell infiltration, especially cytotoxic CD8 T cells, represents superior antitumor ability (50, 51).

Based on the risk score, we further elaborated on TMB, somatic mutations, and CNVs. The high-risk group had a higher TMB, which implied a higher mortality rate. Mutations in TP53 were overwhelmingly predominant in both groups and were more frequent in the high-risk group than in the low-risk group (70% vs. 55%). As previously reported by Lawrence, TP53 mutations and CDKN2 inactivation are intimately involved in HNSCC (52). Remarkably, TP53 mutations are common and associated with a poor prognosis in patients with HNSCC (53). MIR7641 is highly expressed in the exosomes of metastatic tumor cells and can enhance the proliferation, migration, and invasion of recipient tumor cells (54, 55). Cub and Sushi Multiple Domains-1 (CSMD1) acts as a tumor suppressor, whose low expression promotes the invasion of HNSCC and gastric tumor (56, 57) and is also correlated with a poorer prognosis of HNSCC (58).

The efficacy of immunotherapy has been reported to be generally superior to that of conventional chemotherapy (42). Besides, the combined application of PD-1/PD-L1 inhibitors and platinum drugs also shows positive therapeutic potential (59). This implies that the exploration of medication regimens is potentially valuable. On the one hand, the immune checkpoint expressions of LAG3, PDCD1, HAVCR2, CTLA4, and CD274 increased in the low-risk group. The efficacy of corresponding immune checkpoint inhibitors is better for the low-risk group but the opposite for the high-risk group. On the other hand, the low-risk group has a lower TIDE score, which means that the lower TIDE score is related to a better curative effect. TIDE can be used to identify two mechanisms of tumor immune escape: inducing T-cell immunotherapeutic dysfunction in tumors with high infiltration of cytotoxic T lymphocytes (CTLs) and blocking T-cell infiltration in tumors with low CTL in TME (39). In our study, the low-risk group had more infiltration of CTLs, so they would respond better to immunotherapy, due to better recovery from T-cell dysfunction. The high-risk group had less CTL infiltration, so they would benefit less from immunotherapy, which may be due to T-cell repulsion. In short, the low-risk group will benefit more from immunotherapy. Furthermore, we screened out 15 chemotherapy drugs that are more suitable for the high-risk group. The new model constructed by immune-related RBPs could serve as a new marker to help guide the selection of chemotherapeutic drugs and distinguish who would benefit more from antitumor immunotherapy.

Some of the RBPs in this article have been reported to have a practical relationship with tumorigenesis and progression. Asparagine synthetase (ASNS) catalyzes the synthesis of the nonessential amino acid asparagine, while ASNS knockdown significantly hinders cell proliferation (60). In other words, stable ASNS gene expression guarantees the growth of tumor cells. Cortactin (CTTN) gene encodes a protein, cortacn, which plays an essential role in the migration of oral carcinoma cells by regulating filamentous actin and prominent structures on cell membranes (61). The high expression of CTTN was related to a poorer OS rate (62). Coiled-coil-helix-coiled-coil-helix domain-containing protein 2 (CHCHD2) as a small mitochondrial protein can regulate mitochondrial outer membrane permeabilization and is one of the negative regulators that mediate apoptosis (63). CHCHD2 indicates a poor prognosis and is overexpressed in hepatocellular carcinoma, breast tumor, non-small cell lung carcinoma, and renal cell carcinoma (64, 65). The loss of the human Cranio Facial Development Protein 1 (CFDP1) affects the dynamic changes of chromosomes and cell cycle progression (66). Moreover, some studies have confirmed that CFDP1 is a risk gene for pancreatic carcinoma (67, 68). High expression of insulin growth factor 2 mRNA binding protein 1 (IGF2BP1) is associated with a poor prognosis such as advanced clinical stage, increased tumor size, lymph node metastasis, and low survival rate of patients with HNSCC (69, 70). NAD(P)H quinone oxidoreductase (NQO1), a cytoplasmic enzyme that mediates the reduction of quinone substrates, is highly expressed in a multitude of tumors and can catalyze quinone drugs to poison tumor cells (71). NQO1 is considered a promising direct tumor target. For example, the drug β-lapachone, catalyzed by NQO1, triggers the innate perception of T cells in the TME, thereby enhancing antitumor capacity and even overcoming checkpoint blockade (72). Casein kinase 2-interacting protein-1 (CKIP-1, also known as PLEKHO1) inhibits tumor growth by causing inactivation of serine/threonine kinases and self-degradation of Smurf1, which is a potential oncogenic target in various tumor cells (73). Selenium binding protein 1(SELENBP1) is significantly downregulated in esophageal adenocarcinoma, ovarian tumor, and oral squamous cell carcinoma, but its overexpression can lead to incremental cellular senescence and apoptosis, as well as enhanced cytotoxicity of cisplatin (74–76). Three different genes (ATP2A1-3) encode the Ca^2+^-ATPases from the Sarco/endoplasmic reticulum (SERCA) to maintain calcium homeostasis between the cell cytoplasm and the endoplasmic reticulum, and they have been reported to downregulate transcription in gastric and colon tumors (77). In particular, ATP2A2 gene inactivation is closely related to oral squamous cell carcinoma (78). DENN/MADD domain-containing protein 2D (DENND2D) is less expressed in malignant tumors and is thought to contribute to the worsening prognosis and high recurrence rate (79–81). However, other RBPs may have a prospective regulatory impact on HNSCC. Some articles reported the relationship between genes FRMD4A and HNSCC. High expression of FRMD4A is associated with an increased risk of HNSCC recurrence, and the silencing of FRMD4A inhibits the growth and metastasis of human squamous cell carcinoma in skin and tongue metastases and reduces the proliferation and cell adhesion of squamous cell carcinoma (82, 83). Interestingly, in our study, patients with high expression of FRMD4A experienced a better prognosis (**Figure 6H**), which is worthy of further study. RAB proteins play the role of small GTPases in the regulation of vesicle and protein transport, membrane targeting, and fusion, and a group of them can actively or inversely regulate tumor cell generation, migration, and invasion (84). RAB11 affects the invasiveness of breast cancer cells (85). RAB11FIP1 is positively related to dendritic cells and CD4 T cells, and the low expression of RAB11FIP1 revealed a poor prognosis for lung adenocarcinoma (86). CASP8 and FADD-like apoptosis regulator (CFLAR), also known as c-FLICE-like inhibitory protein (c-FLIP), is a vital anti-apoptotic protein (87). Some studies have identified FLIP as an independent poor prognostic indicator for colorectal carcinoma, cervical carcinoma, and acute myeloid leukemia (88).

Although some studies have explored the association of RBPs with HNSCC (89, 90), our research has made further progress. On the basis of differentiated immunophenotyping, we take the lead in the screening of differentially expressed RBPs, which represents a more effective prognostic biomarker and a more accurate predictor of response to immunotherapy in different groups of patients. In general, the prognosis model system constructed based on the immune-related RBPs and clinical information of patients with HNSCC drew the landscape in the immune microenvironment of HNSCC and could effectively predict the prognosis of patients with HNSCC in the high- and low-risk groups. The nomogram based on this model is more helpful for predicting the clinical outcome of patients with HNSCC. Last but not least, the differences in immune checkpoints and TIDE scores between the high- and low-risk groups provide new ideas for the immunotherapy of patients with HNSCC.

Our study still has some limitations. First, we only used public databases to construct and verify the prognostic risk model, and we need to validate this model in subsequent clinical trials. Second, how the immune-related RBPs regulate immunity still needs to be verified by experiments *in vitro* and *in vivo*. Eventually, human papillomavirus is an independent prognostic factor for HNSCC, which is worth further stratified analysis.

## Conclusion

In summary, the signature constructed by 15 immune-related RBPs could effectively predict the clinical outcome of patients with HNSCC. Subsequently, we demonstrated the immune landscape, TMB, CNVs, and efficacy of immunotherapy in different risk groups, which might guide clinical therapy.

## Data Availability Statement

The original contributions presented in the study are included in the article/**Supplementary Material**. Further inquiries can be directed to the corresponding authors.

## Author Contributions

RM and XL designed the study, performed the experiments, and plotted the data. EW collected original data. JW, BL, WY, and PZ drafted and edited the manuscript. HX and SZ reviewed the manuscript. HX and SZ supervised the project. HX and SZ funded the experiments for the study. All authors contributed to the article and approved the submitted version.

## Funding

This study was supported by grants from the National Natural Science Foundation of China (grant numbers 81771002, 82071057).

## Conflict of Interest

The authors declare that the research was conducted in the absence of any commercial or financial relationships that could be construed as a potential conflict of interest.

## Publisher’s Note

All claims expressed in this article are solely those of the authors and do not necessarily represent those of their affiliated organizations, or those of the publisher, the editors and the reviewers. Any product that may be evaluated in this article, or claim that may be made by its manufacturer, is not guaranteed or endorsed by the publisher.
