# Supplementary material for: The Prognostic Signature of Head and Neck Squamous Cell Carcinoma Constructed by Immune-Related RNA-Binding Proteins
Source: Front Oncol. 2022 Apr 5;12:795781. doi: 10.3389/fonc.2022.795781 (PMC9016149; doi:10.3389/fonc.2022.795781)
Supplement: Supplementary file 1 [file Table_1.docx]

**TABLE S1** | 15 immune-related RBP genes and risk coefficient

| **Gene Name** | **Risk coefficient** |
| --- | --- |
| FRMD4A | -0.05735 |
| ASNS | 0.106791 |
| RAB11FIP1 | -0.02582 |
| FAM120C | -0.12207 |
| CFLAR | -0.00259 |
| CTTN | 0.034099 |
| PLEKHO1 | -0.06629 |
| SELENBP1 | -0.01807 |
| CHCHD2 | 0.05469 |
| NPM3 | 0.050738 |
| ATP2A3 | -0.07866 |
| CFDP1 | 0.083365 |
| IGF2BP2 | 0.014861 |
| NQO1 | 0.04586 |
| DENND2D | -0.02068 |
