# Supplementary material for: The Prognostic Signature of Head and Neck Squamous Cell Carcinoma Constructed by Immune-Related RNA-Binding Proteins
Source: Front Oncol. 2022 Apr 5;12:795781. doi: 10.3389/fonc.2022.795781 (PMC9016149; doi:10.3389/fonc.2022.795781)
Supplement: Supplementary file 2 [file Table_2.docx]

**TABLE S2** | Univariate Cox regression analysis of clinical pathologic features TCGA database

| **Variable** | **HR** | **95% Lower CI** | **95% Upper CI** | ***P* value** |
| --- | --- | --- | --- | --- |
| Age | 1.019956 | 1.004066 | 1.036097 | 0.013643 |
| Gender | 1.382316 | 0.966344 | 1.977347 | 0.076307 |
| Grade | 1.17671 | 0.903096 | 1.53322 | 0.228162 |
| Stage | 1.626708 | 1.278764 | 2.069325 | 7.42E-05 |
| T | 1.316171 | 1.103683 | 1.569568 | 0.002227 |
| N | 1.527173 | 1.273161 | 1.831864 | 5.07E-06 |
| RiskScore | 4.456912 | 2.425279 | 8.190424 | 1.48E-06 |
