# Supplementary material for: The Prognostic Signature of Head and Neck Squamous Cell Carcinoma Constructed by Immune-Related RNA-Binding Proteins
Source: Front Oncol. 2022 Apr 5;12:795781. doi: 10.3389/fonc.2022.795781 (PMC9016149; doi:10.3389/fonc.2022.795781)
Supplement: Supplementary file 3 [file Table_3.docx]

**TABLE S3** | Multivariate Cox regression analysis of clinical pathologic features in TCGA database

| **Variable** | **HR** | **95% Lower CI** | **95% Upper CI** | ***P* value** |
| --- | --- | --- | --- | --- |
| Age | 1.02729 | 1.010218 | 1.044651 | 0.001639 |
| Gender | 1.381082 | 0.950521 | 2.006676 | 0.090312 |
| Grade | 1.128149 | 0.845209 | 1.505805 | 0.413095 |
| Stage | 1.473144 | 0.982732 | 2.208285 | 0.060706 |
| T | 0.947057 | 0.73515 | 1.220046 | 0.673807 |
| N | 1.324206 | 1.049387 | 1.670996 | 0.017974 |
| RiskScore | 4.985826 | 2.646216 | 9.393967 | 6.67E-07 |
