# Supplementary material for: The Prognostic Signature of Head and Neck Squamous Cell Carcinoma Constructed by Immune-Related RNA-Binding Proteins
Source: Front Oncol. 2022 Apr 5;12:795781. doi: 10.3389/fonc.2022.795781 (PMC9016149; doi:10.3389/fonc.2022.795781)
Supplement: Supplementary file 4 [file Table_4.docx]

**TABLE S4** | 15 immune-related RBP genes and risk coefficient

| **Gene Name** | **Characteristic** |
| --- | --- |
| FRMD4A | FRMD4A is associated with the growth and metastasis of human squamous cell carcinoma in skin and tongue, while it inhibits the proliferation and cell adhesion of squamous cell carcinoma. |
| ASNS | ASNS catalyzes the synthesis of the nonessential amino acid asparagine, and its knockdown significantly hinders cell proliferation. |
| RAB11FIP1 | RAB proteins play the role of small GTPases in the regulation of vesicle and protein transport, membrane targeting and fusion. RAB11FIP1 is positively related to dendritic cells and CD4 T cells, and its low expression revealed a poor prognosis for lung adenocarcinoma. |
| CFLAR | CFLAR, also known as c-FLIP, is a vital anti-apoptotic protein, which is identified as an independent poor prognostic indicator for colorectal carcinoma, cervical carcinoma and acute myeloid leukemia. |
| CTTN | CTTN encodes protein cortacn, involved in migration of oral carcinoma cell by regulating filamentous actin and prominent structures on cell membranes. Its high expression was related to poorer survival. |
| PLEKHO1 | PLEKHO1 inhibits tumor growth by causing inactivation of serine/threonine kinases and self-degradation of Smurf1, which is a potential oncogenic target in various tumor cells. |
| SELENBP1 | SELENBP1 is significantly down-regulated in esophageal adenocarcinoma, ovarian tumor, and oral squamous cell carcinoma, but its overexpression can lead to incremental cellular senescence and apoptosis, as well as enhanced cytotoxicity of cisplatin. |
| CHCHD2 | CHCHD2 is a small mitochondrial protein can regulate mitochondrial outer membrane permeabilization, and is one of the negative regulators that mediate apoptosis. CHCHD2 indicates a poor prognosis, and is overexpressed in hepatocellular carcinoma, breast tumor, non-small cell lung carcinoma, renal cell carcinoma. |
| ATP2A3 | Three genes (ATP2A1-3) maintain calcium homeostasis between the cell cytoplasm and the endoplasmic reticulum, and they have been reported to down-regulate transcription in gastric and colon tumors. |
| CFDP1 | The loss of the CFDP1 affects the dynamic changes of chromosomes and cell cycle progression. It is a risk gene for pancreatic carcinoma. |
| IGF2BP2 | High expression of IGF2BP1 is associated with advanced clinical stage, increased tumor size, lymph node metastasis and low survival rate of patients with HNSCC. |
| NQO1 | NQO1, a cytoplasmic enzyme that mediates the reduction of quinone substrates, is highly expressed in a multitude of tumors and can catalyze quinone drugs to poison tumor cells. |
| DENND2D | DENND2D is less expressed in malignant tumors and contribute to the poor prognosis and high recurrence rate. |
