# Supplementary figures and images for: The Prognostic Signature of Head and Neck Squamous Cell Carcinoma Constructed by Immune-Related RNA-Binding Proteins
Source: Front Oncol. 2022 Apr 5;12:795781. doi: 10.3389/fonc.2022.795781 (PMC9016149; doi:10.3389/fonc.2022.795781)

A

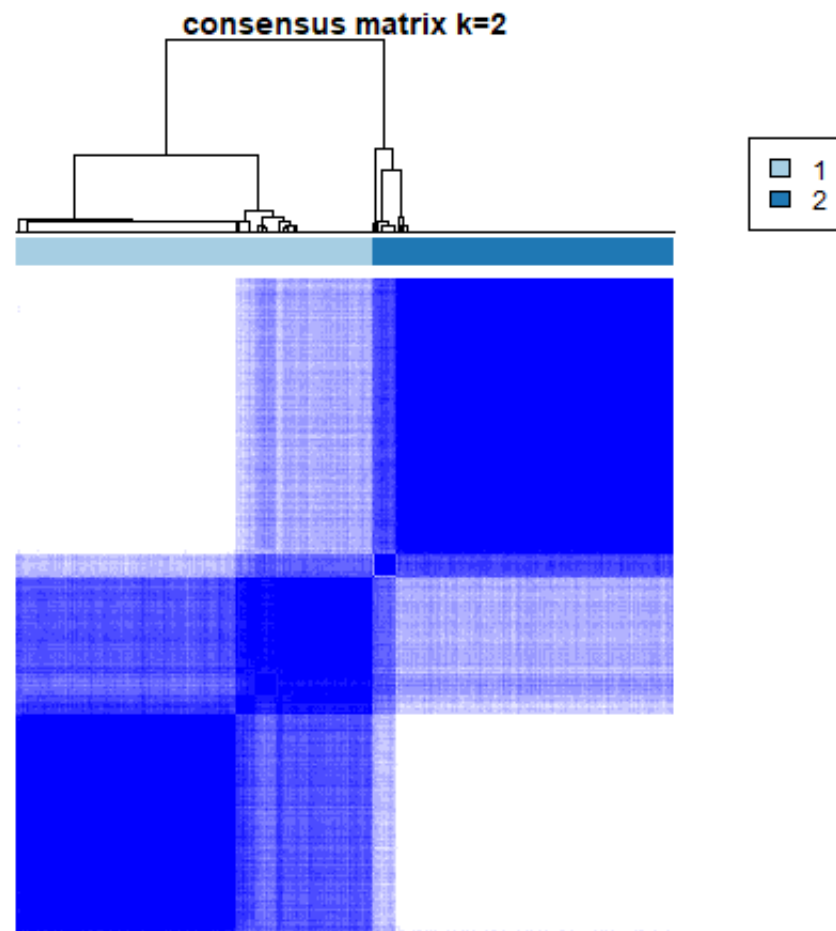

B

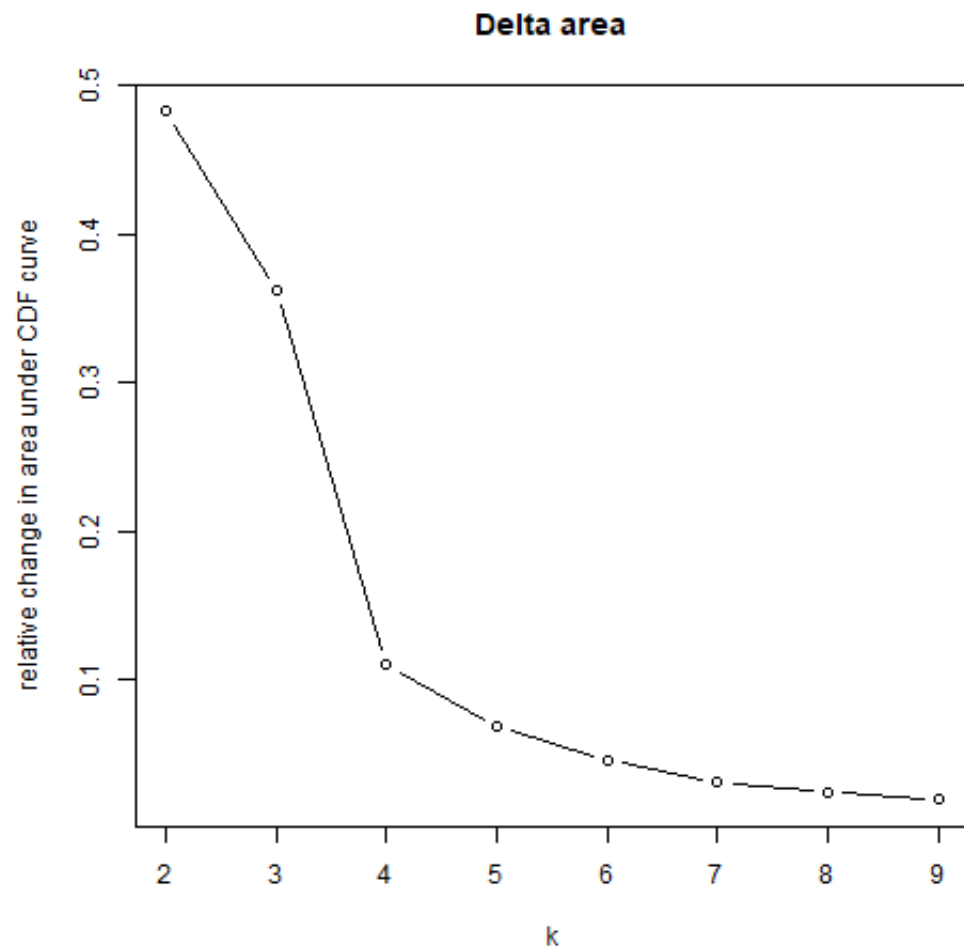

Supplement: Supplementary file 5 [file DataSheet_1.pdf]
